# Incorporating NTCP into Randomized Trials of Proton Versus Photon Therapy

**DOI:** 10.14338/IJPT-18-00038.1

**Published:** 2019-03-21

**Authors:** Jonas Scherman, Ane L. Appelt, Jen Yu, Gitte Fredberg Persson, Lotte Nygård, Johannes A. Langendijk, Søren M. Bentzen, Ivan R. Vogelius

**Affiliations:** 1 Department of Radiation Physics, Skane University Hospital, Lund, Sweden; 2 Department of Oncology, Rigshospitalet, Copenhagen, Denmark; 3 Leeds Institute of Medical Research at St James's, University of Leeds and Leeds Cancer Centre, St James's University Hospital, Leeds, United Kingdom; 4 Department of Radiation Oncology, University of Maryland Medical Center, Baltimore, MD, USA; 5 Department of Radiation Oncology, Miami Cancer Institute, Baptist Health South Florida, Miami, FL, USA; 6 Department of Radiotherapy, University of Groningen, Groningen, The Netherlands; 7 Division of Biostatistics and Bioinformatics, University of Maryland Greenebaum Cancer Center, and Department of Epidemiology and Public Health, University of Maryland School of Medicine, Baltimore, MD, USA

**Keywords:** trial simulation, trial design, lung cancer, proton therapy

## Abstract

**Purpose:**

We propose and simulate a model-based methodology to incorporate heterogeneous treatment benefit of proton therapy (PrT) versus photon therapy into randomized trial designs. We use radiation-induced pneumonitis (RP) as an exemplar. The aim is to obtain an unbiased estimate of how predicted difference in normal tissue complications probability (ΔNTCP) converts into clinical outcome on the patient level.

**Materials and Methods:**

ΔNTCP data from in silico treatment plans for photon therapy and PrT for patients with locally advanced lung cancer as well as randomly sampled clinical risk factors were included in simulations of trial outcomes. The model used at point of analysis of the trials was an iQUANTEC model. Trial outcomes were examined with Cox proportional hazards models, both in case of a correctly specified model and in a scenario where there is discrepancy between the dose metric used for ΔNTCP and the dose metric associated with the “true” clinical outcome, that is, when the model is misspecified. We investigated how outcomes from such a randomized trial may feed into a model-based estimate of the patient-level benefit from PrT, by creating patient-specific predicted benefit probability distributions.

**Results:**

Simulated trials showed benefit in accordance with that expected when the NTCP model was equal to the model for simulating outcome. When the model was misspecified, the benefit changed and we observed a reversal when the driver of outcome was high-dose dependent while the NTCP model was mean-dose dependent. By converting trial results into probability distributions, we demonstrated large heterogeneity in predicted benefit, and provided a randomized measure of the precision of individual benefit estimates.

**Conclusions:**

The design allows for quantifying the benefit of PrT referral, based on the combination of NTCP models, clinical risk factors, and traditional randomization. A misspecified model can be detected through a lower-than-expected hazard ratio per predicted ΔNTCP.

## Introduction

Radiation oncology has—as have many other technology-driven medical disciplines—struggled to generate level I evidence demonstrating the clinical benefits of technologic advances. This is also the case for proton therapy [1], where the superiority over standard photon therapy remains controversial [2–4], even for widely accepted treatments such as pediatric malignancies [5–7]. It has been suggested that purely relying on evidence created from randomized controlled trials (RCTs) may not be the optimal way of identifying patients (or patient groups) likely to benefit from proton therapy [8–11]. An alternative methodology for development of data-based treatment strategies involves prediction of individual patient benefit using normal tissue complication probability (NTCP) models [8]. The health care payers in some European countries have accepted NTCP model predictions as a basis for reimbursement of the cost of proton therapy [8, 12]. Still, selection of patients, based on outcome prediction models, suffers from risk of bias or inaccurate results if the underlying model lacks in accuracy or generalizability.

From an evidence-based medicine perspective, proton and photon therapies should be compared in RCTs, as would be required for any novel drug, except in indications where the benefit is deemed to be so large that randomization as a method for avoiding bias may not be necessary [13, 14]. At the time of writing, a number of clinical trials of proton therapy are indeed in progress in a wide range of indications [15]. The challenge of this approach is that simple head-to-head comparison ignores the expected heterogeneity of treatment effect: Some patients may expect large benefits from proton therapy in terms of toxicity reduction, while others may have relatively little to gain (or might even be disadvantaged). Or to put it differently: an RCT of proton versus photon therapy may provide a methodologically correct answer to the wrong question—whether protons are uniformly better than photons in a defined population of patients—while missing out on the more relevant question—who, if any, among the patients will have a clinically meaningful benefit from proton therapy compared with photon therapy.

Here, we propose and simulate an approach where the heterogeneous treatment benefit of proton therapy predicted by a model is incorporated into the trial design. This idea also extends into the recurrent discussions regarding personalized medicine [16], where technology-driven improvements have the potential to play a major role. We show how such a randomized trial may provide a randomized estimate of the patient-level benefit from proton therapy, using comparative dose planning and taking clinical risk factors for toxicity into account.

As a proof-of-concept, we demonstrate the design and interpretation of a hypothetical trial of definitive radiation therapy for locally advanced non–small cell lung cancer (NSCLC), with radiation-induced pneumonitis (RP) as the primary endpoint. The model used at point of analysis of the trials is an individualized QUANTEC (iQUANTEC) model taking clinical risk factors into account [17, 18].

## Materials and Methods

### Dose Planning

Twenty consecutive patients with locally advanced NSCLC treated with definitive radiation therapy and concomitant chemotherapy at our center, from January 1, 2015, and onwards, were selected as dose-planning cases, to provide realistic estimates of interpatient heterogeneity in dose metric differences. This retrospective study was approved by the Danish Health Authority, approval No. 3-3013-817/1, in accordance with Danish law. All patients were treated in free breathing with volumetric modulated arc therapy. Robust proton dose plans were generated, and clinical photon plans and generated proton dose plans fulfilled clinical constraints. See Supplementary Materials for additional information on patient data, dose planning, and clinical constraints.

### Dose-Volume Histogram Data

For each dose plan (photon and proton) the mean lung dose (MLD) and the volumes receiving at least 10 Gy, 20 Gy, 30 Gy, 40 Gy, and 60 Gy (V_10Gy_, V_20Gy_, V_30Gy_, V_40Gy_, and V_60Gy_) were retrieved for the lungs (total lung minus gross tumor volume). Mean heart dose and V_35Gy_ to esophagus were also retrieved. Data were retrieved with Eclipse Scripting API (version 13.6) using in-house developed software (Visual Studio Community Edition 2015).

**Figure 1:**
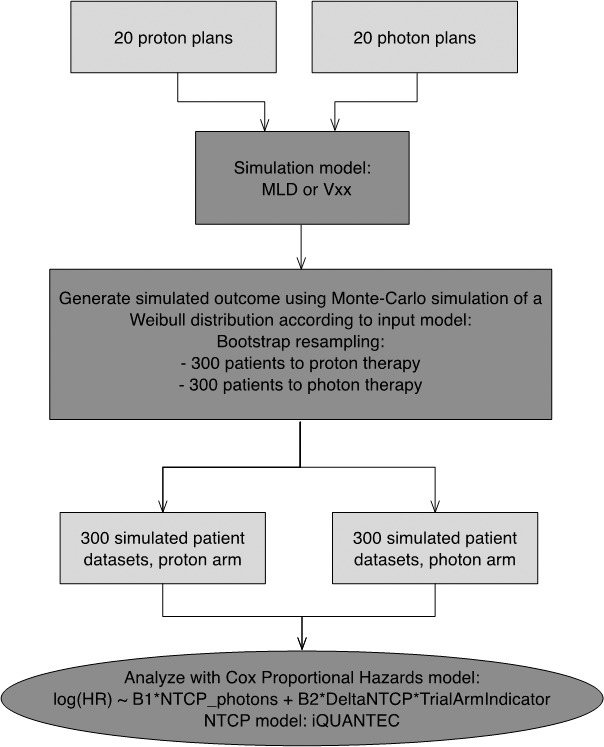
Schematics of the trial simulation setup. Twenty patients with dual planning provided a distribution of dose plans. These dose plans were input into a simulation model, which used either the iQUANTEC/QUANTEC model based on mean lung dose or a V_XX_ model to simulate a baseline risk of toxicity corresponding to a given dose distribution. These data were forwarded to a simulation stage, where the dose plans were sampled with replacement for up to 2 × 300 simulated trial patients. Each patient also had a randomly generated set of clinical risk factors (smoking, pulmonary comorbidity, and age), and the combination of the radiation dose and clinical risk factors could then be used to generate a patient-specific Weibull distribution of event probability versus follow-up time. Simulated binary survival data were generated from the corresponding probabilities. The result was a 2 × 300 patient data structure simulating clinical outcomes in a trial. This “in silico trial dataset” was analyzed according to our suggested strategy: using the complication probability of an individualized QUANTEC NTCP model as a covariate in a Cox model (see figure for the exact expression used in the model). Abbreviations: HR, hazard ratio; MLD, mean lung dose; NTCP, normal tissue complication probability.

We considered the setting of a randomized trial of proton versus photon therapy for locally advanced NSCLC, with rate of symptomatic RP as primary endpoint and with a 1:1 allocation ratio to the 2 trial arms and 300 patients per arm. **Figure 1** shows the overall simulation setup.

**Figure 2:**
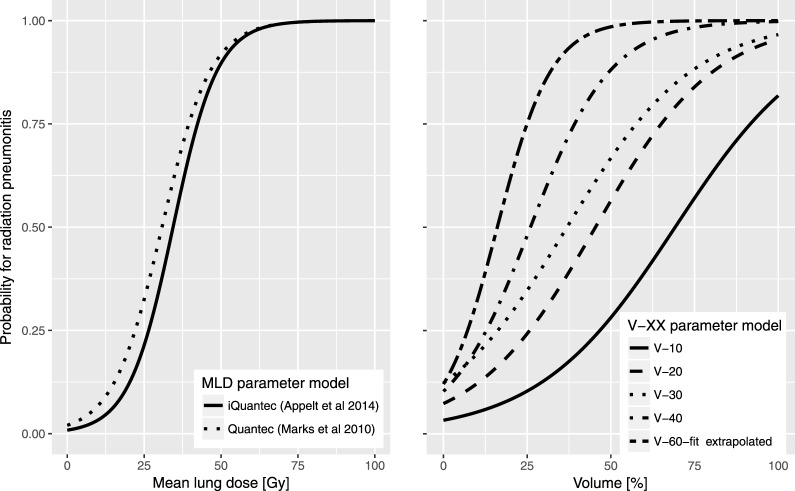
MLD dose-risk models (left) from Marks et al [19] and Appelt et al [18]. Alternative dose-risk models (right) for the dose-volume histogram parameters used in simulation of outcomes (first dark grey box in Figure 1) from Willner et al [21]. Abbreviation: MLD, mean lung dose.

We assumed the risk of RP could be predicted by the dose plan MLD and calculated an individual patient risk factor, *NTCP_sum_*, for RP, using an individualized QUANTEC model (iQUANTEC) [18]. This model uses the dose-response relationship for symptomatic RP found in the QUANTEC review [19], but integrates additional clinical risk factors identified in a literature-based meta-analysis and their associated odds ratios (ORs) [20] (see **Figure 2**). We assumed a logistic dose-response relationship and estimated the OR for RP compared to a reference (see below) for a patient with a specific *MLD* and a set of clinical risk factors *X_1_, X_2_*..., as

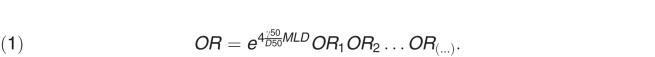



A summarized individual patient risk was estimated by using the logarithm of the *OR*, *NTCP_sum_* = *ln(OR)*, as this is additive in changes in risk factors.

We simulated outcomes by using a Weibull hazard function, *h(t),* parameterized by the shape and scale parameters *ρ* and *λ*:

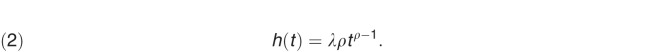



Individual changes in the estimated risk of event (RP) were modeled by adjusting the scale parameter *λ_risk_ = λexp(NTCP_sum_) = λOR*. Further, we required a patient with baseline clinical risk factors and average MLD with photon therapy (17.0 Gy) to have a probability of freedom from RP at 2 years of 85%, which in turn defines *ρ = 0.6* and *λ = 0.11*. Finally, we assumed follow-up evenly distributed over a 2-year period.

For simulation of trial outcomes, we used bootstrap resampling from the 20 patients with dual planning. For each sample, we used the photon dose plan MLD for risk estimation based on dose and randomly sampled clinical risk factors as identified by Vogelius and Bentzen [20], with prevalences as summarized in the study of Appelt et al [18]: preexisting pulmonary comorbidity, OR 2.27, prevalence 0.26; mid or inferior tumor location, OR 1.87, prevalence 0.44; current smoker, OR 0.62, prevalence 0.28; age >63 years, OR 1.66, prevalence 0.50.

To include the effect of the experimental treatment (proton therapy), we calculated the individual change in risk (ie, Δ*NTCP*) resulting only from the reduction in NTCP by using the difference in MLD between photon and proton therapy. We randomly selected patients for the experimental arm and adjusted their individual risk factor according to: *NTCP_sum.proton_ = NTCP_sum.photon_* + Δ*NTCP*. Event times were simulated by sampling from the corresponding Weibull distribution (ie, with *λ_risk_*, based on either *NTCP_sum.proton_* or *NTCP_sum.photon_*, as appropriate), and censoring times were randomly sampled from a uniform distribution over the interval of 0 to 2 years.

Once the simulated trial dataset was created, we used the Cox proportional hazards model to analyze the data. The ΔNTCP and NTCP_sum.photons_ were always included as covariates in the Cox model (**Figure 1**).

### Model Misspecification

Model misspecification refers to the scenario where the user is making NTCP and ΔNTCP estimates (eg, in the model-based selection process) based on a certain parametrization (eg, MLD), but where the driver of toxicity is another metric (eg, lung V_40Gy_).

**Figure 3:**
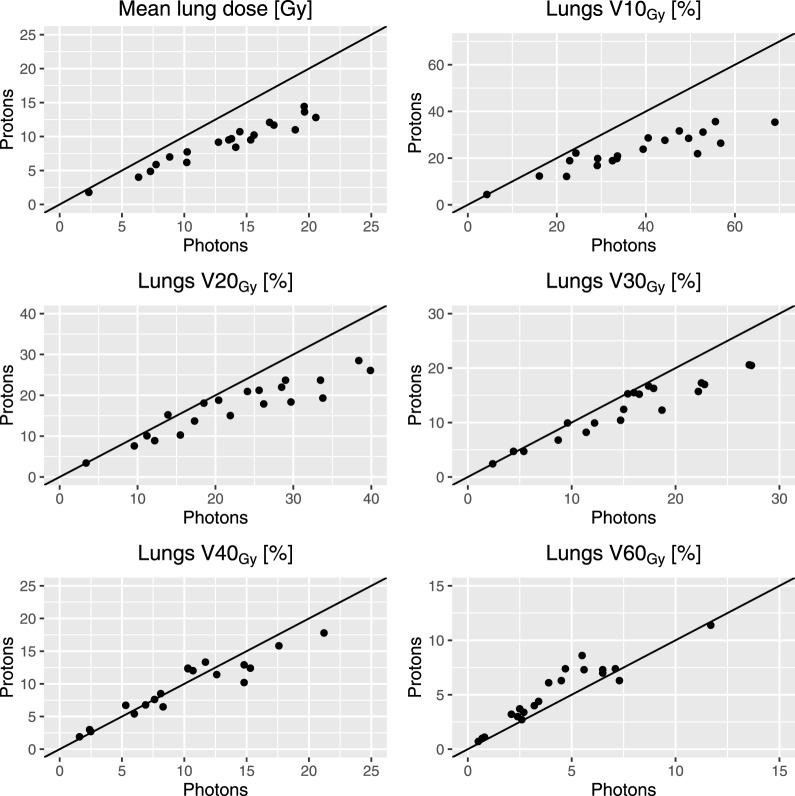
Dosimetric comparison of the 20 patients' photon (x-axis) and proton (y-axis) doses to the lung structure (lungs minus gross tumor volume). The identity line indicates where the 2 modalities yielded same results, and points below the identity line indicate superiority of protons as compared to photons and vice versa. Note the different scales on both the x- and y-axis.

To study the effect of model misspecification, we changed the dose-volume model used in the simulation of outcome data while keeping the analysis of trial data the same. The assumed alternative driver dose-response models were based on dose cutoffs (V_XX_-driven models) and were taken from literature data [21]. These models did not account for clinical risk factors and were not modified further. Fitting a logistic model to the data set provided estimates of γ_50_ and D_50_ (See Supplementary Materials). As Willner et al [21] only included predictive parameters up to V_40Gy_, the data were extrapolated to V_60Gy_ in order to test a dose metric that correlated differently with MLD in the photon and proton plans (**Figure 3**). Supplementary Materials tabulate γ_50_ and D_50_ estimates for the different models; see **Figure 2** for graphical versions of all models.

For both the main analysis (Trial Simulation) and the model misspecification we simulated 1000 trials each with 2 × 300 patients and report the resulting estimate of the logarithm of the hazard ratio [log(HR)] per ΔNTCP from the Cox model. Log(HR) per ΔNTCP is calculated by assuming the QUANTEC model for MLD (receiver model) regardless of which model was used to drive the simulated trial outcomes. To assess the effect of uncertainty associated with delivery of proton therapy on the trial outcome, we also included a worst-case proton plan MLD, selected from the uncertainty calculations performed during the planning process (using uncertainties of 0.5-cm isocenter shift and 3.5% calibration curve error). The worst-case proton plan was defined as the uncertainty plan with the highest MLD and was used as alternative driver dose-response models.

### Application of Trial Results in Selecting Patients for Referral

Finally, we wanted to illustrate how the outcome of a trial could be used to support clinical decisions for future patients. The most frequent log(HR) per ΔNTCP, from 1000 simulations with a correctly specified model, was used for illustration. The trial yielded a log(HR) per ΔNTCP (based on a randomized comparison) for protons versus photons and we converted this result into an estimate of the expected benefit in 4 illustrative cases: the patient with the highest ΔMLD and a patient with approximately median ΔMLD, in both cases assuming no clinical risk factors or *all* clinical risk factors present (note that “current smoker” has a protective effect, ie, OR < 1). The effect size estimate of the Cox proportional hazards model could be used to provide a probability distribution of absolute benefit from proton therapy at the individual patient level, using the basic properties of the Cox model: 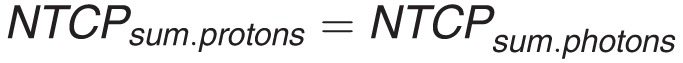
^*HR*^, where HR = exp(β*ΔNTCP) and β [=log(HR)] were assumed normally distributed according to the confidence interval of the simulated trial. This approach naturally combines the model prediction (NTCP) and the result of randomization, as log(HR) is an estimate with ΔNTCP as covariable in strict randomization.

All simulations and analysis of outcome data were performed in RStudio (RStudio: Integrated Development for R, RStudio Team [2015], RStudio Inc, Boston, Massachusetts).

## Results

### Dose Plan Comparison

The mean ΔMLD was 4.3 Gy when comparing the robust planned proton dose plans with the clinical photon dose plans (**Figure 3**). The proton dose plans had a lower MLD and a lower V_20Gy_ for all patients. However, V_40Gy_ and V_60Gy_ were higher with protons in an increasing proportion of cases. Evaluating the worst-case proton plans, the mean ΔMLD was 3.2 Gy.

### Trial Simulation

**Figure 4:**
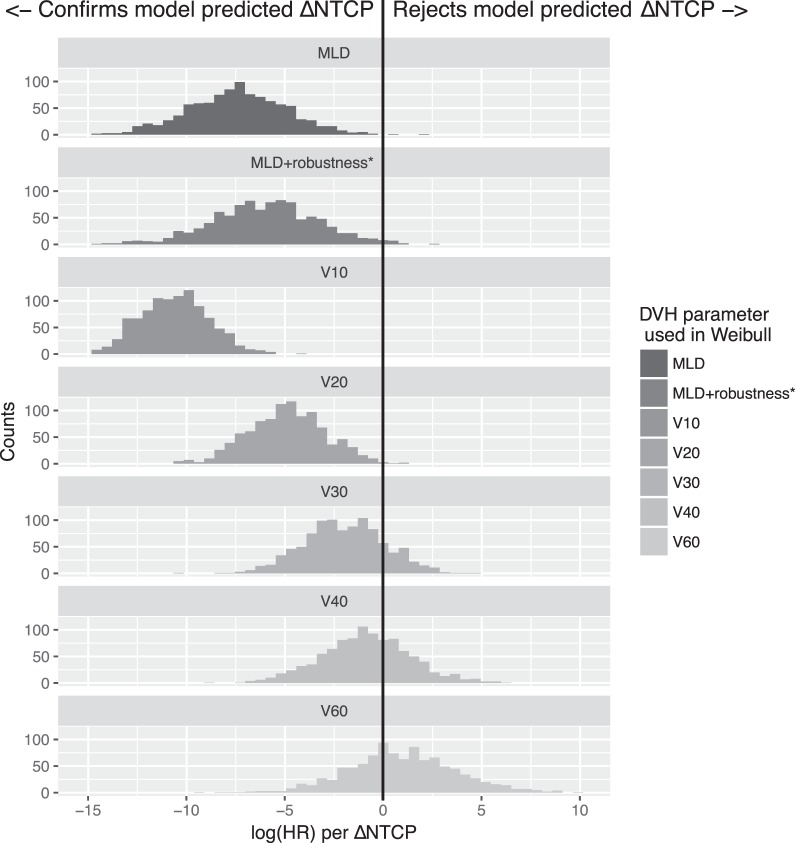
Simulations of 1000 trials for each volumetric parameter, according to the scheme in Figure 1 for 2 × 300 patients. In the top set, the model is correctly specified (MLD used for simulating outcome and MLD used in Cox regression analysis of results). On the x-axis is log(HR) per ΔNTCP of the Cox proportional hazards model. As expected, when the MLD model is used to generate data, the outcomes favor the model-predicted ΔNTCP [log(HR) < 0]. When the model is misspecified, the outcome favors protons even more for V_10Gy_ as the underlying model, and progressively moves towards favoring photons for the V_60Gy_ model as input (ie, HR per ΔNTCP favors the plan with highest NTCP in the model). Comparing with Figure 3, this illustrates that the trials will reject a benefit of treating with protons if V_60Gy_ is the underlying driver of RP, as desired. *MLD+robustness assumes the worst MLD taken from proton uncertainty plans, using uncertainties of 0.5-cm isocenter shift and 3.5% calibration curve error. Abbreviations: DVH, dose-volume histogram; HR, hazard ratio; MLD, mean lung dose; NTCP, normal tissue complication probability; RP, radiation-induced pneumonitis.

The simulated trials favored proton therapy when an input MLD-based model was used (correctly specified model), and progressively decreased the predicted benefit of protons when the input model was changed from V_10Gy_ towards V_60Gy_ (**Figure 4**). Assuming the worst-case MLD (MLD-robustness in **Figure 4**) from the proton uncertainty plans had a limited effect on the trial outcome.

### Use in Future Patients

**Figure 5:**
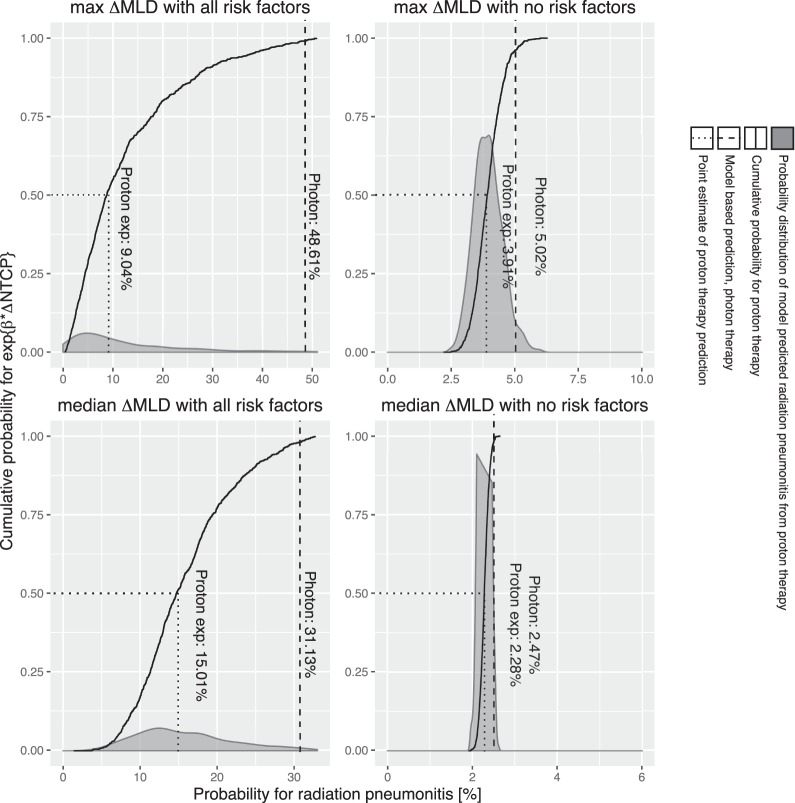
Application of hypothetical trial outcome for a future patient. Top row: A patient with photon MLD = 18.9 Gy and proton MLD = 11.0 Gy (highest ΔMLD among the 20 patients). Top left shows the case where this patient has all risk factors (ie, a patient with preexisting pulmonary comorbidity, mid or inferior tumor location, old age, and not a current smoker) present (50% chance of improving NTCP from 48.6% to less than 9.2%); and top right shows the case where no risk factors (no comorbidities, upper tumor location, young age, and current smoker) are present (50% chance of improving from 5.0% to 3.9%). Bottom row: A patient with photon MLD = 13.6 Gy and proton MLD = 9.5 Gy (median ΔMLD among the 20 patients). Bottom left shows the case where this patient has all risk factors present and bottom right shows the case where no risk factors are present. Abbreviations: MLD, mean lung dose; NTCP, normal tissue complication probability; Proton exp, proton experimental arm.

We finally assumed the completion of a trial with a resulting estimate of the value of log(HR) for ΔNTCP. If the model was well specified, the most frequent trial result was used: log(HR) per ΔNTCP = −7.77 (95% confidence limit: −16.6 to 1.05). This result was converted into probability distributions of predicted benefit, shown for 4 illustrative cases in **Figure 5**, detailing how a patient with all risk factors in combination with a large ΔMLD reduction, comparing photons to protons, will have a reduced probability of developing RP, namely, from 49% to 9%. Additionally, a patient with the same number of risk factors, but median ΔMLD between the 2 modalities, will only have a 31% to 15% probability reduction.

## Discussion

Our trial simulation incorporates heterogeneity of treatment effect and quantified risk at the individual patient level into a randomized comparison of 2 treatment strategies. The aim is to obtain an unbiased estimation of how a difference in model-predicted ΔNTCP converts into a clinical outcome on the patient level.

**Figure 5** shows how outcomes from randomized trial results can be used to quantify the individual benefits of proton versus photon therapy and thereby support the decision to refer to proton therapy on a rational basis. This allows moving from simple hypothesis testing in a comparative effectiveness trial towards individualized estimates of benefit. Arguably, the cases presented in **Figure 5** reflect subjective decisions already made by treating physicians, but the estimate of the HR per ΔNTCP quantifies the relative merits of the 2 radiation modalities and provides decision support for patients, caregivers, and policy makers. A supportive decision-making tool based on the data from this article has been developed in RStudio and is available online at: https://protontrialsimulation.shinyapps.io/trial_webb/.

The trial design presented here still requires strict randomization. A possible elaboration on the present approach could be to use adaptive Bayesian designs. In such a design patients with a large predicted benefit are randomly assigned with higher weighting to proton therapy and when a predefined limit on the magnitude of benefit is exceeded, the patient can be allocated to the according arm without randomization at all.

A major strength of the presented design is that future selection of patients does not rely on the model being correctly specified. The estimate of HR per ΔNTCP adjusts the predicted benefit to the actual clinical data and thus reduces the risk of wrongly allocating future patients to protons if, for example, the V_60Gy_ model would be a better predictor of NTCP. In other words, where the model-based approach relies on nonrandomized follow-up studies after implementation to identify model misspecification, the current proposal detects model misspecification from randomized data through the HR per ΔNTCP from a trial. Another strength of the design is that an uncertainty in the MLD did not affect the trial outcome (**Figure 4**).

It should be acknowledged that it is not ideal only to consider NTCP of a single endpoint in benefit estimations. Here we focused on a single endpoint for simplicity, but further studies should look at several NTCP endpoints, preferably at a more comprehensive NTCP profile as pointed out by others [11], but also include verification that the tumor control probability is not affected by choice of modality.

To illustrate this point in the lung cancer radiation therapy setting, we considered acute esophagitis and heart complications, both of which are of high relevance. We provide NTCP estimates for acute esophagus [22] and heart toxicity [23] in the Supplementary Materials. A reduction in NTCP for the heart when using protons compared to photons was observed, but we did not see a reduction in the risk of acute esophagitis (Supplementary Materials).

Evaluating only a reduction in MLD, the expected difference in RP in an RCT should favor intensity-modulated proton therapy. A recent study from MD Anderson Cancer Center (Houston, Texas) on an RCT comparing modulated photon therapy and passive scattering proton therapy in lung cancer [4] indicated that there was no difference in either MLD or RP rates between the 2 arms. However, the authors noted a difference in RP between early and late inclusion in the proton group, with no difference in MLD. The fact that the late proton group had both a significant smaller tumor volume and lower delivered dose might explain the lower RP rate. This suggests that higher prescribed doses further elucidate the risk of a higher-than expected dose metric from the lung and might itself be associated with the probability of RP induction when using proton therapy.

## Conclusion

Our proposed trial design quantifies the benefit of referral to proton therapy, based on the combination of NTCP models and traditional randomization. The proposed method behaves well under the investigated types of NTCP model misspecification and provides quantitative benefit estimates on the individual patient level, which may have greater clinical utility than the information derived from a traditionally designed RCT.
