# Supplementary material for: Incorporating NTCP into Randomized Trials of Proton Versus Photon Therapy
Source: Int J Part Ther. 2019 Mar 21;5(3):24–32. doi: 10.14338/IJPT-18-00038.1 (PMC6874185; doi:10.14338/IJPT-18-00038.1)
Supplement: Supplementary file 1 [file ijpt-05-03-05_s01.docx]

**Supplementary Materials for:** Incorporating NTCP into randomized trials of proton versus photon therapy

Patient information is presented in Table A1.

Table A1: Patient information table.

| Patient # | Total GTV volume  [cm^3^] | Total CTV volume  [cm^3^] | Total PTV volume [cm^3^] | Tumor motion measured on 4DCT  AP, CC, LR  [mm] | CTV to PTV margin calculated according to Josipovic et al [1]  AP CC LR  [mm] |
| --- | --- | --- | --- | --- | --- |
| 1 | 177 | 319 | 548 | 5, 7, 2 | 7, 7, 5 |
| 2 | 32 | 96 | 224 | 2, 1, 1 | 6, 6, 5 |
| 3 | 71 | 141 | 314 | 5, 5, 3 | 7, 6, 5 |
| 4 | 4 | 17 | 51 | 1, 1, 1 | 6, 6, 5 |
| 5 | 5 | 56 | 146 | 2, 4, 2 | 6, 6, 5 |
| 6 | 234 | 366 | 608 | 4, 3, 3 | 6, 6, 5 |
| 7 | 99 | 169 | 320 | 2, 2, 2 | 6, 6, 5 |
| 8 | 132 | 235 | 439 | 2, 2, 2 | 6, 6, 5 |
| 9 | 116 | 243 | 533 | 1, 8, 6 | 6, 7, 6 |
| 10 | 81 | 159 | 292 | 2, 2, 2 | 6, 6, 5 |
| 11 | 49 | 102 | 196 | 2, 5, 2 | 6, 6, 5 |
| 12 | 4 | 19 | 70 | 7, 7, 1 | 7, 7, 5 |
| 13 | 42 | 113 | 280 | 2, 12, 4 | 6, 8, 5 |
| 14 | 185 | 295 | 476 | 1, 2, 1 | 6, 6, 5 |
| 15 | 12 | 46 | 145 | 2, 7, 0 | 6, 7, 5 |
| 16 | 263 | 368 | 567 | 1, 1, 1 | 6, 6, 5 |
| 17 | 136 | 244 | 404 | 2, 2, 2 | 6, 6, 5 |
| 18 | 56 | 172 | 318 | 3, 2, 5 | 7, 6, 5 |
| 19 | 188 | 316 | 557 | 3, 8, 4 | 7, 7, 5 |
| 20 | 45 | 134 | 328 | 3, 10, 3 | 7, 8, 5 |

GTV – gross tumor volume, CTV – clinical target volume, PTV – planning target volume. AP – anterior posterior, CC – Cranio caudal, LR – Left right.

## Constraints used for treatment planning

Dose limiting constraints used during the dose planning process for both photon and proton dose plans are shown in Table A2. Spinal cord, lungs, esophagus and heart were considered as organs at risk.

Table A2: Dose limiting constraints used for dose planning. PRV – Planning organs at risk volume.

| **Organ** | **Max Constraint** | **Volume constraint** | **Mean Constraint** |
| --- | --- | --- | --- |
| Spinal cord | < 45 Gy_RBE_ | - | - |
| PRV Spinal cord | < 50 Gy_RBE_ | - | - |
| Lungs minus gross tumor volume | - | V_20 GyRBE_ < 35% and V_60 GyRBE_ < 5% | < 20 Gy_RBE_ |
| Esophagus | < 66 Gy_RBE_ | V_70 GyRBE_ < 1 cc | - |
| Heart | - | V_50 GyRBE_ < 20% | < 46 Gy_RBE_ |

## Photon dose plans

Dose planning was performed in Eclipse v13.0 (Varian Medical Systems, Palo Alto, USA) on a mid-ventilation computer tomography (CT) image set selected from a 4DCT [2] using VMAT with two half arcs to 66 Gy_RBE_ in 2 Gy_RBE_ fractions. Individual margins for the expansion from clinical target volume (CTV) to planning target volume (PTV) were based on motion during a pre-treatment 4DCT [2] and calculated according to Josipovic et al [1]. Photon dose plans were normalized to achieve 100% mean dose to the PTV. Photon dose plans were calculated using the Anisotropic Analytical Algorithm version 13.0 (Varian Medical Systems, Palo Alto, CA, USA).

## Proton dose plans

Proton dose planning was performed retrospectively in Eclipse v13.6, on the same CT-image and structure sets used for photon dose planning with a clinical pencil beam scanning proton beam model. Two or three treatment fields (depending on the anatomical location of the target) were applied with a snout position of 10 cm for all fields. Single field optimization was used to achieve clinical constraints and coverage of the target volumes with the same prescription as the photon dose plan (66 Gy_RBE_ in 2 Gy_RBE_ fractions, using a relative biological effectiveness of 1.1). Clinical optimization constraints were identical to the photon dose plan. Dose plan normalization was identical to the one for the photon dose plans using the same PTV, prescribing 100% mean dose to the PTV, to allow for a reasonable comparison between modalities. The distal and proximal margins were set individually for each field based on the proximal and distal distance between the PTV and field entrance, accounting for the total distance and using a 3.5% uncertainty of the Hounsfield units (HU) to relative stopping power conversion. The average proximal and distal margins were 0.34 and 0.72 cm with ranges of 0.2 to 0.5 cm and 0.5 to 1.1 cm, respectively. Lateral field margins were set to 1.0 cm. A standard calibration curve between relative stopping power and HU was used. The robustness of the proton plans was validated after optimization to ensure 95% coverage of the CTV using uncertainties of 0.5 cm isocenter shift and 3.5% calibration curve error. Proton dose plans were calculated using the Proton Convolution Superposition algorithm, version 13.6 (Varian Medical Systems, Palo Alto, CA, USA).

## NTCP models for V_xx_ parameters

The volumetric response parameters for RP probability were taken from Willner et al [3], where values for V_10Gy_, V_20Gy_, V_30Gy_ and V_40Gy_ were extracted from Figure 4 (Willner et al [3]) and fitted with a logistic function according to:

$NTCP=\frac{1}{1+exp\{4\gamma_{50}(1-\frac{V_{XX}}{D_{50}})\}}$ .

Data for V_60Gy_ were retrieved by extrapolating data from V_10Gy_ to V_40Gy_, and model parameters estimated by fitting the extrapolated data to the same logistic function. The parameters γ_50_ and D_50_ for all models are presented in Table A2 with their respective references.

Table A3: Parameters for logistic models for different dose volume histogram parameters, used as input parameters in the Weibull simulations. V_xx_ parameters were retrieved using an in-house developed curve fitting software. DVH – dose volume histogram, MLD – mean lung dose.

| **DVH-parameter in RP model** | **γ_50_** | **D_50_ [Gy]** | **From reference** |
| --- | --- | --- | --- |
| V_10Gy_ | 0.85 | 69.19 | Willner et al [3] |
| V_20Gy_ | 0.63 | 45.39 | Willner et al [3] |
| V_30Gy_ | 0.49 | 36.90 | Willner et al [3] |
| V_40Gy_ | 0.54 | 25.98 | Willner et al [3] |
| V_60Gy_ – extrapolated and fitted with logistic function | 0.50 | 16.08 | Extrapolated from Willner et al [3] |
| MLD with risk factors | 0.97 | 30.8 | Marks et al [4] |
| MLD with no risk factors | 1.19 | 34.4 | Appelt et al [5] |

## NTCP for heart and esophagus

For the two other common organ complications after radiotherapy, coronary complications [6] and esophageal toxicity, we estimated the expected complication rate based on the in-silico dose plans. For acute esophageal toxicity ≥ grade 2, we used a model for esophageal toxicity taken from Belderbos et al [7]. For risk of radiotherapy-induced major coronary event, we used the model from Darby et al [6]. The results of those estimates suggest that there is essentially no potential gain in NTCP reduction of esophageal toxicity, but a clear potential reduction in complications for the heart when using protons compared to photons.


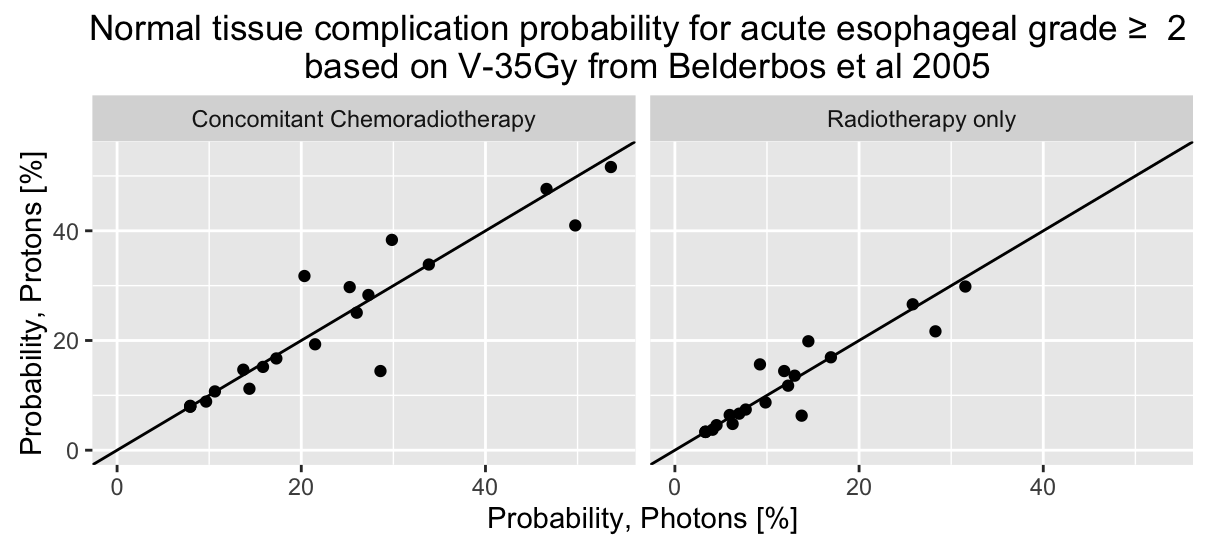


Figure A1: Normal tissue complication probability for acute esophageal grade 2 based on V_35Gy_ calculated according to Belderbos et al [7]. Solid lines indicates a slope of unity.


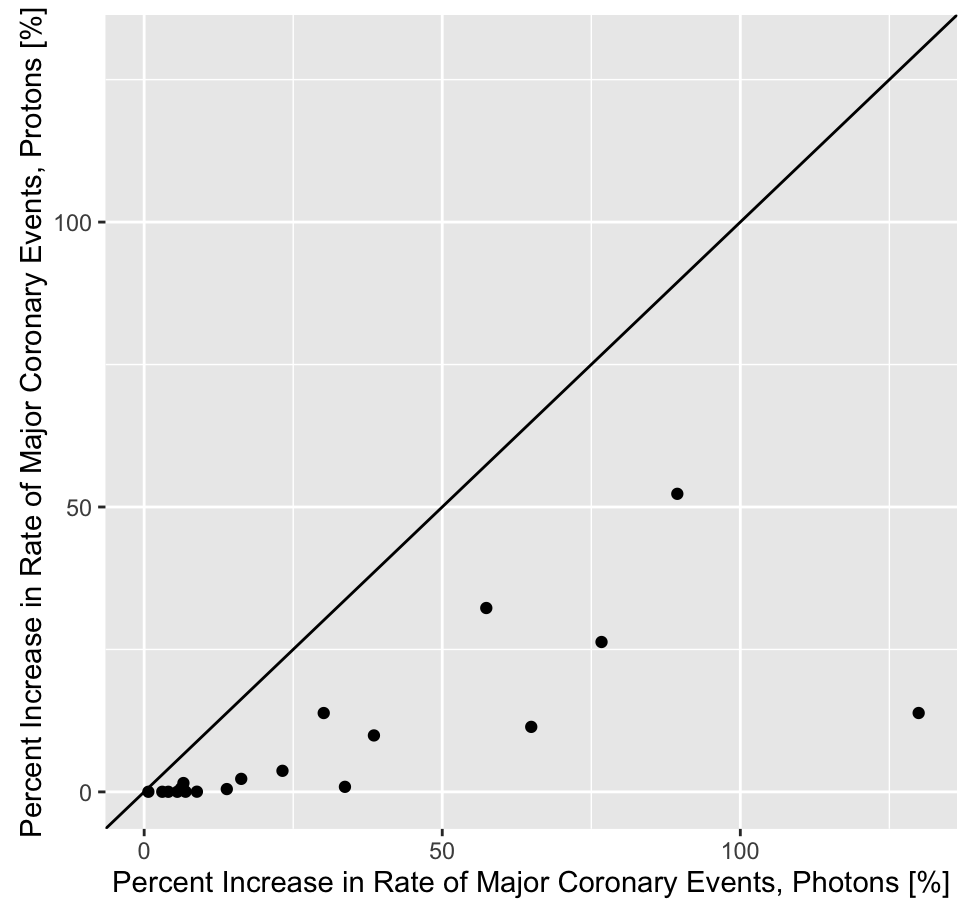


Figure A2: Percent increase in rate of major coronary events for photons vs protons calculated according to Darby et al [6]. Solid line indicates a slope of unity.

# References

1. Josipovic M, Persson GF, Dueck J, Bangsgaard JP, Westman G, Specht L, Aznar MC. Geometric uncertainties in voluntary deep inspiration breath hold radiotherapy for locally advanced lung cancer. Radiother Oncol. 2015;

2. Wolthaus JW, Schneider C, Sonke JJ, van Herk M, Belderbos JS, Rossi MM, Lebesque J V, Damen EM. Mid-ventilation CT scan construction from four-dimensional respiration-correlated CT scans for radiotherapy planning of lung cancer patients. Int J Radiat Oncol Biol Phys. 2006;65:1560–71.

3. Willner J, Jost A, Baier K, Flentje M. A little to a lot or a lot to a little? An analysis of pneumonitis risk from dose-volume histogram parameters of the lung in patients with lung cancer treated with 3-D conformal radiotherapy. Strahlentherapie und Onkol. 2003;179:548–56.

4. Marks LB, Bentzen SM, Deasy JO, Kong F-M (Spring), Bradley JD, Vogelius IS, et al. Radiation Dose–Volume Effects in the Lung. Int J Radiat Oncol Biol Phys. 2010;76:S20–7.

5. Appelt AL, Vogelius IR, Farr KP, Khalil A a, Bentzen SM. Towards individualized dose constraints: Adjusting the QUANTEC radiation pneumonitis model for clinical risk factors. Acta Oncol. 2014;53:605–12.

6. Darby SC, Ewertz M, McGale P, Bennet AM, Blom-Goldman U, Bronnum D, Correa C, Cutter D, Gagliardi G, Gigante B, Jensen MB, Nisbet A, Peto R, Rahimi K, Taylor C, Hall P. Risk of ischemic heart disease in women after radiotherapy for breast cancer. N Engl J Med. 2013;368:987–98.

7. Belderbos J, Heemsbergen W, Hoogeman M, Pengel K, Rossi M, Lebesque J. Acute esophageal toxicity in non-small cell lung cancer patients after high dose conformal radiotherapy. Radiother Oncol. 2005;75:157–64.
